# Complete chloroplast genome of *Artemisia schmidtiana* Maxim. (Asteraceae) and its phylogenetic placement

**DOI:** 10.1080/23802359.2026.2711168

**Published:** 2026-07-30

**Authors:** Youming Ai, Zhiyun Lin, Tianlai Huang, Zixuan Wang, Linmei Ye

**Affiliations:** ^a^ College of Agriculture and Biotechnology, Lishui University, Lishui, China; ^b^ Jinyun Xiandu Middle School, Lishui, China; ^c^ Qingtian County Forestry Technology Extension Station, Lishui, China

**Keywords:** Anthemideae, organellar genomics, Illumina sequencing

## Abstract

*Artemisia schmidtiana* Maxim. is a perennial species in the family Asteraceae. We assembled and characterized its complete chloroplast genome using Illumina sequencing data. The *A. schmidtiana* plastome is 151,032 bp long and has an overall GC content of 37.45%. The plastome contains 111 unique genes, comprising 79 protein-coding genes, 28 tRNA genes, and 4 rRNA genes. Phylogenetic analysis of 71 shared chloroplast protein-coding genes placed *A. schmidtiana* within the sampled *Artemisia* taxa and close to *A. tournefortiana*. This plastome provides a genomic resource for molecular identification, comparative plastome studies, and phylogenetic analyses of *Artemisia*.

## Introduction

*Artemisia schmidtiana* Maxim. 1872 is a low-growing, rhizomatous perennial species of *Artemisia* L. (Asteraceae). The species is naturally distributed from the southeastern Russian Far East and Sakhalin to Japan, where it occurs mainly in the temperate biome (POWO 2026). In Japan, it has been recorded from open habitats, including coastal sites and bare ground in high mountains (Ohwi 1965). *Artemisia schmidtiana* forms compact silver-gray mounds with finely divided, lightly aromatic leaves and is widely cultivated as an ornamental foliage plant for rock gardens, borders, and ground-cover plantings (Royal Horticultural Society 2026). The genus *Artemisia* is one of the largest and most taxonomically complex groups in the tribe Anthemideae, comprising numerous medicinal, aromatic, ecological, and ornamental species distributed mainly across temperate regions of the Northern Hemisphere (Bremer and Humphries 1993; Watson et al. 2002). Previous molecular studies have shown that the infrageneric classification and phylogenetic relationships of *Artemisia* and its allied genera remain complex, partly because of extensive morphological variation and close interspecific relationships (Torrell et al. 1999; Watson et al. 2002).

Chloroplast genomes are generally conserved in structure and gene content among angiosperms and have been widely used for plant species identification, comparative genomics, and phylogenetic reconstruction (Wicke et al. 2011; Dong et al. 2015; Daniell et al. 2016). Complete plastome sequences can provide more informative characters than a single or a few DNA loci, especially for resolving relationships among closely related species. Although plastome resources have increasingly been used in phylogenetic studies of Asteraceae and *Artemisia*, the complete chloroplast genome of *A. schmidtiana* has not been described in detail. This plastome can help document genomic variation in a horticulturally used but poorly sampled *Artemisia* species and provide a reference for species identification, comparative plastome analysis, and phylogenetic placement within the genus. Here, we assembled and annotated the complete chloroplast genome of *A. schmidtiana* and investigated its phylogenetic position within *Artemisia*.

## Materials and methods

**Figure 1.**
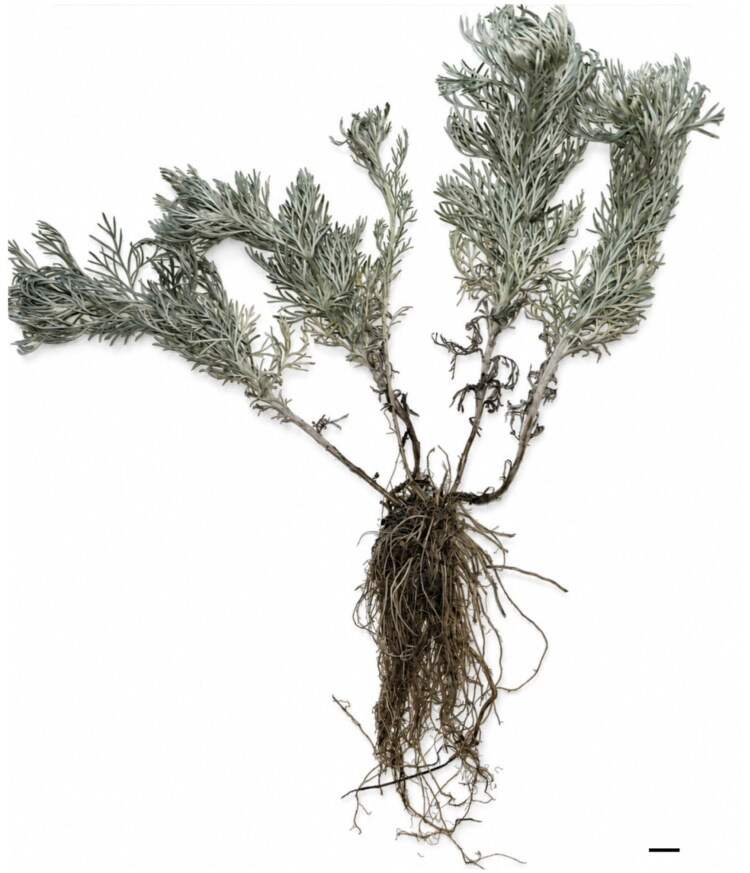
*Artemisia schmidtiana*. Freshly collected whole plant at the vegetative stage before pressing. Photograph taken by Youming Ai. Scale bar = 1 cm.

Plant material of *A. schmidtiana* was collected on 3 June 2025 from an open urban green space in Sanhe Subdistrict, Xindu District, Chengdu, Sichuan Province, China (30°45′57″N, 104°07′08″E). The photographed specimen was in the vegetative growth stage, and no reproductive structures were present at collection. Youming Ai identified the specimen based on a low tufted perennial habit, dense pubescence, silver-gray foliage, and leaves dissected into narrow linear lobes, which are diagnostic vegetative characters consistent with published descriptions of *A. schmidtiana* (Maximowicz 1872; Ohwi 1965). A voucher specimen was deposited in the Herbarium of Lishui University, Lishui, Zhejiang, China, under the provisional institutional herbarium acronym LISHUI and voucher number LISHUI-2025-06-016 (contact: Linmei Ye, linmeiye_qt@126.com; Figure 1).

Genomic DNA was extracted from leaves using a Rapid Plant Genomic DNA Isolation Kit (Sangon Biotech, Shanghai, China). Library preparation and sequencing were performed by Sangon Biotech (Shanghai, China). A paired-end genomic DNA library was constructed using the TruSeq DNA Sample Preparation Kit (Illumina, San Diego, CA, USA), and sequencing was performed on the Illumina HiSeq 2500 platform to generate 150-bp paired-end reads. Raw paired-end reads were assessed and filtered using fastp v0.20.1 (Chen et al. 2018) with default quality-filtering settings and eight threads. Adapter-containing reads and reads with excessive ambiguous bases were removed or trimmed. The clean reads were then assembled into a complete chloroplast genome using GetOrganelle v1.8.0.1 (Jin et al. 2020). The organelle type was set to embplant_pt, extension rounds were set to 15 (-R 15), k-mer sizes were set to 21, 45, 65, 85, and 105 (-k 21,45,65,85,105), and eight threads were used (-t 8). Except for these options, default settings were used. The complete command was: get_organelle_from_reads.py −1 As.clean_1.fastq.gz −2 As.clean_2.fastq.gz -o getorganelle_pt -F embplant_pt -R 15 -k 21,45,65,85,105 -t 8. The fastp command was: fastp -i As.raw_1.fastq.gz -I As.raw_2.fastq.gz -o As.clean_1.fastq.gz -O As.clean_2.fastq.gz -h fastp.html -j fastp.json -w 8.

Coverage depth of the assembled genome was calculated with SAMtools v1.16.1 (Li et al. 2009), and the depth/coverage profile was visualized using ggplot2 v3.3.5 in R v4.1.2 (R Core Team 2021; Wickham 2016; Figure S1). The assembled chloroplast genome had an average sequencing depth of 377.64× and a minimum depth of 2×; all sites were covered by at least one read, and 99.995% of sites were covered at ≥10× depth. Annotation was performed using GeSeq (Tillich et al. 2017) and CPGAVAS2 (Shi et al. 2019), which were used to determine gene start positions, inverted repeat (IR) boundaries, and other gene loci. CPGView (Liu et al. 2023) was then used to refine the annotation, visualize the chloroplast genome architecture, and characterize cis-splicing and trans-splicing gene structures.

The complete chloroplast genome of *A. schmidtiana*, together with 31 representative *Artemisia* plastomes, was included in the phylogenetic analysis, with *Ajania tenuifolia* as the outgroup. GenBank accession numbers for all taxa are listed in Table S1. Shared chloroplast protein-coding genes were extracted from GenBank-format plastome files using Biopython v1.79 (Cock et al. 2009). Seventy-one protein-coding genes shared by all taxa were retained. Each gene was aligned separately using MAFFT v7.490 (Katoh and Standley 2013). The alignments were trimmed using ClipKIT v2.12.0 in smart-gap mode with codon-aware trimming (Steenwyk et al. 2020) and concatenated into a final matrix of 60,324 aligned nucleotide positions. Maximum-likelihood phylogenetic reconstruction was performed using IQ-TREE v3.1.2 (Minh et al. 2020). The best-fit nucleotide substitution model was selected by ModelFinder according to the Bayesian information criterion, and TVM+F + I + R3 was selected as the optimal model (Kalyaanamoorthy et al. 2017). Branch support was assessed using 1000 ultrafast bootstrap replicates (Hoang et al. 2018). The final tree was rooted with *A. tenuifolia*.

## Results

**Figure 2.**
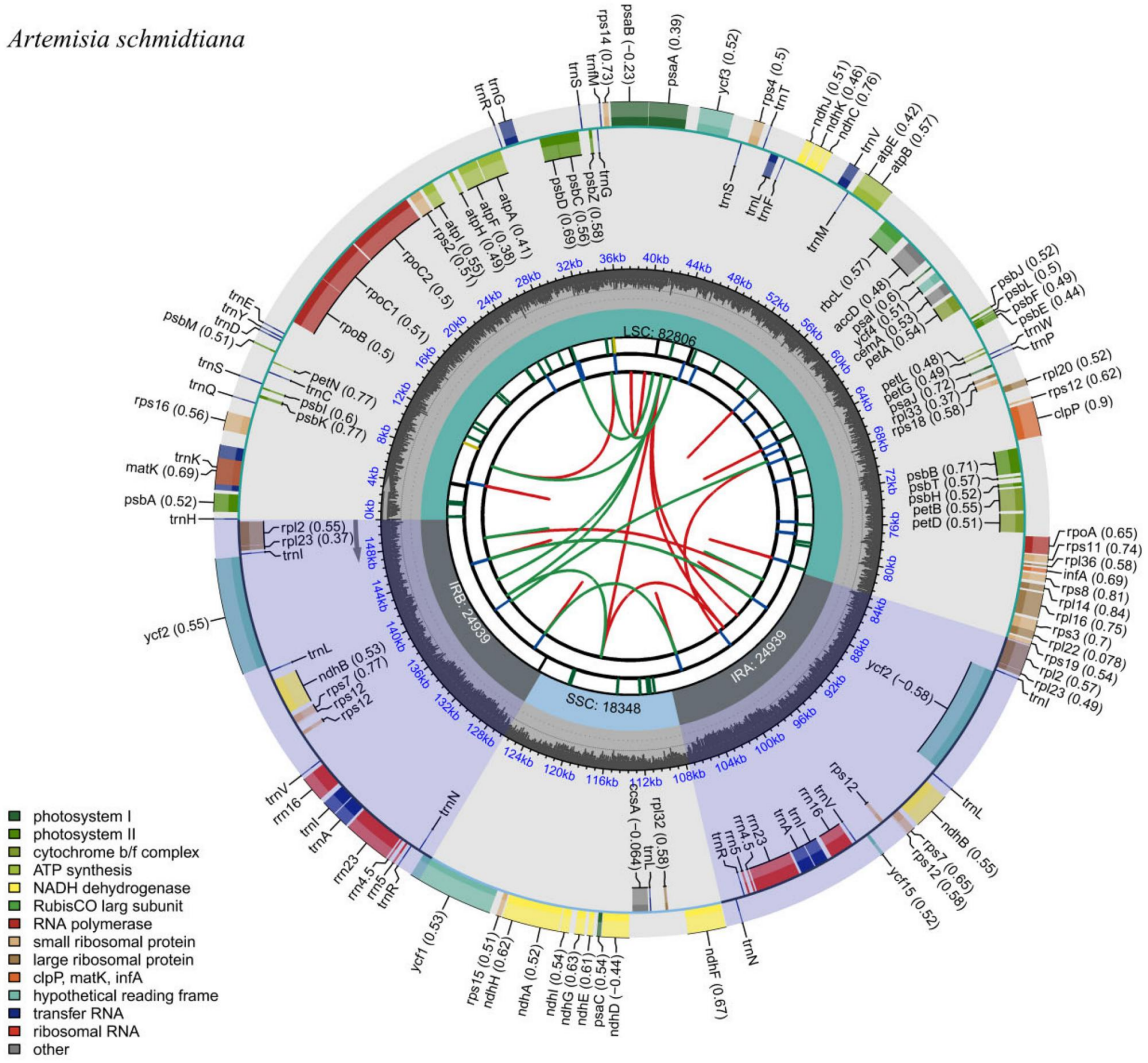
Plastome map of *Artemisia schmidtiana*. The inner circle shows GC content (dark grey) and AT content (light grey). Genes on the outer circle are color-coded by functional category.

The complete chloroplast genome of *A. schmidtiana* was 151,032 bp long and had an overall GC content of 37.45%. The plastome showed the typical quadripartite structure, consisting of an 82,806 bp large single-copy (LSC) region, two 24,939-bp inverted repeats (IRa and IRb), and an 18,348 bp small single-copy (SSC) region (Figure 2). Genome annotation identified 111 unique genes, including 79 protein-coding genes, 28 transfer RNA genes, and 4 ribosomal RNA genes. Gene composition is summarized in Table S2, and the complete gene annotation, including genomic coordinates, intron status, and IR duplication, is provided in Table S4. Cis-splicing analysis identified 13 cis-spliced gene copies representing 11 unique genes: *rps16*, *rpoC1*, *atpF*, *ycf3*, *clpP*, *petB*, *petD*, *rpl16*, *rpl2*, *ndhB*, and *ndhA*. Among these, *rpl2* and *ndhB* were duplicated in the IR regions (Figure S2). The *rps12* gene showed a characteristic trans-spliced structure, with exon 1 located in the LSC region and duplicated exon 2/exon 3 copies distributed in the IR regions (Figure 2; Figure S3). A comparison of plastome features between *A. schmidtiana* and nine *Artemisia* plastomes is provided in Table S3.

**Figure 3.**
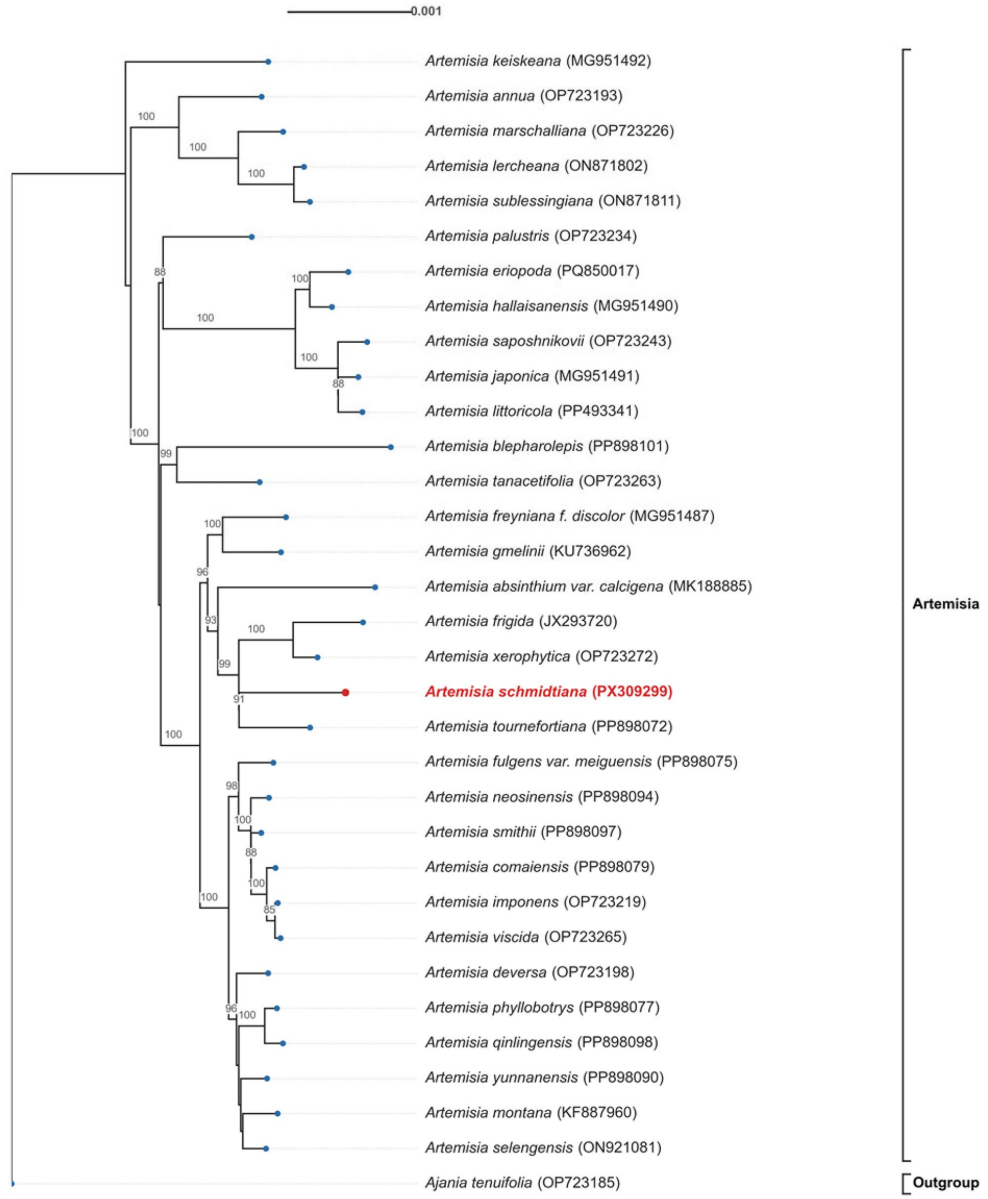
Maximum-likelihood phylogenetic tree inferred from 71 shared chloroplast protein-coding genes of 32 *Artemisia* taxa, with *Ajania tenuifolia* used as the outgroup. Node labels indicate ultrafast bootstrap support values. *Artemisia schmidtiana* is highlighted in red. The scale bar represents substitutions per site. The following sequences were used: *Artemisia keiskeana* MG951492 (Kim et al. 2020), *A. annua* OP723193 (unpublished), *A. marschalliana* OP723226 (unpublished), *A. lercheana* ON871802 (unpublished), *A. sublessingiana* ON871811 (unpublished), *A. palustris* OP723234 (unpublished), *A. eriopoda* PQ850017 (unpublished), *A. hallaisanensis* MG951490 (Lim et al. 2018), *A. saposhnikovii* OP723243 (unpublished), *A. japonica* MG951491 (Kim et al. 2020), *A. littoricola* PP493341 (Kadam et al. 2024), *A. blepharolepis* PP898101 (unpublished), *A. tanacetifolia* OP723263 (unpublished), *A. freyniana* f. *discolor* MG951487 (Kim et al. 2020), *A. gmelinii* KU736962 (Lee et al. 2016), *A. absinthium* var. *calcigena* MK188885 (Shahzadi et al. 2020), *A. frigida* JX293720 (Liu et al. 2013), *A. xerophytica* OP723272 (unpublished), *A. schmidtiana* PX309299 (this study), *A. tournefortiana* PP898072 (unpublished), *A. fulgens* var. *meiguensis* PP898075 (unpublished), *A. neosinensis* PP898094 (unpublished), *A. smithii* PP898097 (unpublished), *A. comaiensis* PP898079 (unpublished), *A. imponens* OP723219 (unpublished), *A. viscida* OP723265 (unpublished), *A. deversa* OP723198 (unpublished), *A. phyllobotrys* PP898077 (unpublished), *A. qinlingensis* PP898098 (unpublished), *A. yunnanensis* PP898090 (unpublished), *A. montana* KF887960 (unpublished), *A. selengensis* ON921081 (Wang et al. 2024), and *Ajania tenuifolia* OP723185 (unpublished).

The maximum-likelihood tree was reconstructed from a 60,324-nucleotide trimmed concatenated matrix of 71 shared chloroplast protein-coding genes and included 32 *Artemisia* taxa plus *Ajania tenuifolia* as the outgroup (Figure 3). In the rooted tree, *A. schmidtiana* was placed within the sampled *Artemisia* plastomes and close to *A. tournefortiana*. The topology was therefore interpreted only with respect to the phylogenetic placement of *A. schmidtiana* within the sampled taxa.

## Discussion and conclusion

Comparative plastome data place *A. schmidtiana* within the structural range observed across the nine *Artemisia* plastomes summarized in Table S3, including the closely related *A. frigida*, *A. gmelinii*, and *A. freyniana*. Across the sampled taxa, total plastome size varied by only 286 bp (151,032–151,318 bp), whereas IR length varied by only 46 bp (24,939-24,985 bp). Thus, the small differences in total genome size were concentrated mainly in the single-copy regions, particularly the LSC region, rather than reflecting pronounced IR expansion or contraction. Together with the similar GC contents (37.42-37.49%), these patterns indicate that the plastome architecture of *A. schmidtiana* has remained structurally stable relative to the sampled congeners. In Asteraceae, IR expansion and contraction are important sources of plastome-size and gene-duplication variation (Wicke et al. 2011; Daniell et al. 2016); the nearly invariant IR lengths observed here instead suggest limited recent IR-boundary displacement within the sampled *Artemisia* lineage.

The minor variation in annotated unique gene number (111–114) should be interpreted cautiously because differences in annotation practice can affect counts of small or duplicated genes. Nevertheless, the shared core gene complement, conserved intron-containing genes, and stable IR-associated duplicated genes, including *rpl2*, *ndhB*, and the trans-spliced *rps12*, support strong conservation of the plastid genetic system. Repetitive sequences are potential substrates for local indels and intramolecular recombination in plastomes; however, the absence of large regional length shifts or changes in the duplicated IR gene set suggests that repeat-associated variation in the sampled taxa has not produced detectable large-scale structural reorganization. Such variation is therefore more likely to contribute to fine-scale sequence polymorphism than to major plastome architectural divergence. This interpretation is consistent with previous comparative studies of *Artemisia* plastomes (Kim et al. 2020) and supports a conservative mode of plastome evolution within the sampled *Artemisia* taxa.

The plastome-based phylogeny placed *A. schmidtiana* close to *A. tournefortiana* within the sampled *Artemisia* taxa. As plastid genomes are typically maternally inherited in angiosperms, this topology reflects the evolutionary history of the plastid lineage rather than the complete species history (Birky 2001). Nuclear genomic data with broader taxon and population sampling will be needed to test the relationships among *A. schmidtiana*, *A. tournefortiana*, and *A. frigida* and to evaluate potential effects of hybridization or incomplete lineage sorting within *Artemisia*. Because the analysis included one non-*Artemisia* outgroup and a limited subset of *Artemisia* species, the topology should not be interpreted as a test of generic monophyly. Evaluating the monophyly of *Artemisia* would require broader sampling of closely related genera and additional appropriate outgroups. Although no independent DNA barcode-based pre-verification was performed, the plastome placement was consistent with the morphology-based identification of the voucher specimen. Given the taxonomic complexity of *Artemisia*, which is associated with morphological similarity, hybridization, and polyploidy, this plastome sequence provides a useful genomic resource for species identification, comparative plastome analysis, and future evolutionary studies of the genus.

In conclusion, this study reports the complete chloroplast genome of *A. schmidtiana* and its placement within a plastome-based phylogeny of sampled *Artemisia* taxa. The plastome had a conserved quadripartite structure and contained 111 unique genes. These data expand chloroplast genomic resources for *Artemisia* and provide a reference for molecular identification and phylogenetic studies of this taxonomically complex genus.

## Supplementary Material

Supplemental Material.docx

## Data Availability

The genome sequence generated in this study is openly available from NCBI GenBank under accession number PX309299. The associated BioProject, SRA, and BioSample numbers are PRJNA1303833, SRR34948206, and SAMN50537045, respectively.
