# Supplementary material for: Complete chloroplast genome of Artemisia schmidtiana Maxim. (Asteraceae) and its phylogenetic placement
Source: Mitochondrial DNA B Resour. 2026 Jul 30;11(9):985–90. doi: 10.1080/23802359.2026.2711168 (PMC13425525; doi:10.1080/23802359.2026.2711168)
Supplement: Supplemental Material.docx [file TMDN_A_2711168_SM0267.docx]

**Complete chloroplast genome of *Artemisia schmidtiana* Maxim. (Asteraceae) and its phylogenetic placement**

Youming Ai, Zhiyun Lin, Tianlai Huang, Zixuan Wang, Linmei Ye

**Figure S1.** **The distribution of chloroplast genome sequencing depth for *Artemisia schmidtiana* is depicted in the graph, with the horizontal axis representing genomic position and the vertical axis indicating sequencing depth.**

**Figure S2.** **The cis-splicing genes in the chloroplast genome of *Artemisia schmidtiana* are depicted in a schematic map using CPGView.** The exons of the cis-splicing genes are represented in black, while the introns are shown in white. Arrows indicate the sense direction of genes. It should be noted that the lengths of exons and introns are not drawn to scale.

**Figure S3.** **The trans-spliced gene *rps12* in the chloroplast genome of *Artemisia schmidtiana* is depicted in a schematic map using CPGView.** An arrow indicates the sense direction of the gene. It should be noted that the lengths of exons are not drawn to scale.

**Table S1. Chloroplast genome sequences used for phylogenetic analysis.**

| **Taxon** | **GenBank accession** | **Reference / source** | **Role** |
| --- | --- | --- | --- |
| *Artemisia keiskeana* | MG951492 | Kim et al., 2020 | Ingroup |
| *Artemisia annua* | OP723193 | GenBank (reference not specified) | Ingroup |
| *Artemisia marschalliana* | OP723226 | GenBank (reference not specified) | Ingroup |
| *Artemisia lercheana* | ON871802 | GenBank (reference not specified) | Ingroup |
| *Artemisia sublessingiana* | ON871811 | GenBank (reference not specified) | Ingroup |
| *Artemisia palustris* | OP723234 | GenBank (reference not specified) | Ingroup |
| *Artemisia eriopoda* | PQ850017 | GenBank (reference not specified) | Ingroup |
| *Artemisia hallaisanensis* | MG951490 | Lim et al., 2018 | Ingroup |
| *Artemisia saposhnikovii* | OP723243 | GenBank (reference not specified) | Ingroup |
| *Artemisia japonica* | MG951491 | Kim et al., 2020 | Ingroup |
| *Artemisia littoricola* | PP493341 | Kadam et al., 2024 | Ingroup |
| *Artemisia blepharolepis* | PP898101 | GenBank (reference not specified) | Ingroup |
| *Artemisia tanacetifolia* | OP723263 | GenBank (reference not specified) | Ingroup |
| *Artemisia freyniana f. discolor* | MG951487 | Kim et al., 2020 | Ingroup |
| *Artemisia gmelinii* | KU736962 | Lee et al., 2016 | Ingroup |
| *Artemisia absinthium var. calcigena* | MK188885 | Shahzadi et al., 2020 | Ingroup |
| *Artemisia frigida* | JX293720 | Liu et al., 2013 | Ingroup |
| *Artemisia xerophytica* | OP723272 | GenBank (reference not specified) | Ingroup |
| *Artemisia schmidtiana* | PX309299 | This study | Ingroup |
| *Artemisia tournefortiana* | PP898072 | GenBank (reference not specified) | Ingroup |
| *Artemisia fulgens var. meiguensis* | PP898075 | GenBank (reference not specified) | Ingroup |
| *Artemisia neosinensis* | PP898094 | GenBank (reference not specified) | Ingroup |
| *Artemisia smithii* | PP898097 | GenBank (reference not specified) | Ingroup |
| *Artemisia comaiensis* | PP898079 | GenBank (reference not specified) | Ingroup |
| *Artemisia imponens* | OP723219 | GenBank (reference not specified) | Ingroup |
| *Artemisia viscida* | OP723265 | GenBank (reference not specified) | Ingroup |
| *Artemisia deversa* | OP723198 | GenBank (reference not specified) | Ingroup |
| *Artemisia phyllobotrys* | PP898077 | GenBank (reference not specified) | Ingroup |
| *Artemisia qinlingensis* | PP898098 | GenBank (reference not specified) | Ingroup |
| *Artemisia yunnanensis* | PP898090 | GenBank (reference not specified) | Ingroup |
| *Artemisia montana* | KF887960 | GenBank (reference not specified) | Ingroup |
| *Artemisia selengensis* | ON921081 | Wang et al., 2024 | Ingroup |
| *Ajania tenuifolia* | OP723185 | GenBank (reference not specified) | Outgroup |

**Table S2. Gene composition in the chloroplast genome of *Artemisia schmidtiana*.**

| **Category** | **Gene Group** | **Gene Name** | **Number** |
| --- | --- | --- | --- |
| Photosynthesis | Subunits of photosystem I | *psaA, psaB, psaC, psaI, psaJ* | 5 |
|  | Subunits of photosystem II | *psbA, psbB, psbC, psbD, psbE, psbF, psbH, psbI, psbJ, psbK, psbL, psbM, psbT, psbZ* | 14 |
|  | Subunits of NADH dehydrogenase | *ndhA*, ndhB*, ndhC, ndhD, ndhE, ndhF, ndhG, ndhH, ndhI, ndhJ, ndhK* | 12 |
|  | Subunits of cytochrome b6/f complex | *petA, petB*, petD*, petG, petL, petN* | 6 |
|  | Subunits of ATP synthase | *atpA, atpB, atpE, atpF*, atpH, atpI* | 6 |
|  | Large subunit of rubisco | *rbcL* | 1 |
| Self-replication | Proteins of large ribosomal subunit | *rpl14, rpl16*, rpl2*, rpl20, rpl22, rpl23, rpl32, rpl33, rpl36* | 11 |
|  | Proteins of small ribosomal subunit | *rps11, rps12**, rps14, rps15, rps16*, rps18, rps19, rps2, rps3, rps4, rps7, rps8* | 14 |
|  | Subunits of RNA polymerase | *rpoA, rpoB, rpoC1*, rpoC2* | 4 |
|  | Ribosomal RNAs | *rrn16, rrn23, rrn4.5, rrn5* | 8 |
|  | Transfer RNAs | *trnA-UGC*, trnC-GCA, trnD-GUC, trnE-UUC, trnF-GAA, trnG-UCC*, trnH-GUG, trnI-CAU, trnI-GAU*, trnK-UUU*, trnL-CAA, trnL-UAA*, trnL-UAG, trnM-CAU, trnN-GUU, trnP-UGG, trnQ-UUG, trnR-ACG, trnR-UCU, trnS-GCU, trnS-GGA, trnS-UGA, trnT-UGU, trnV-GAC, trnV-UAC*, trnW-CCA, trnY-GUA, trnfM-CAU* | 36 |
| Other genes | Maturase | *matK* | 1 |
|  | Protease | *clpP*** | 1 |
|  | Envelope membrane protein | *cemA* | 1 |
|  | Acetyl-CoA carboxylase | *accD* | 1 |
|  | c-type cytochrome synthesis gene | *ccsA* | 1 |
|  | Translation initiation factor | *infA* | 1 |
| Genes of unknown function | Conserved hypothetical chloroplast ORF | *ycf1, ycf15, ycf2, ycf3**, ycf4* | 6 |

A total of 111 unique genes were annotated, including 79 protein-coding genes, 28 tRNA genes, and 4 rRNA genes. *Gene with one intron; **gene with two introns or trans-splicing. The number column indicates annotated gene copies, including duplicated copies in the IR regions.

**Table S3. Comparative chloroplast genome features of *Artemisia schmidtiana* and nine *Artemisia* species**

| **Species** | **Accession** | **Genome size (bp)** | **GC (%)** | **LSC (bp)** | **IR (bp)** | **SSC (bp)** | **Unique genes (PCGs/tRNAs/rRNAs)** |
| --- | --- | --- | --- | --- | --- | --- | --- |
| *Artemisia schmidtiana* | PX309299 | 151,032 | 37.45 | 82,806 | 24,939 | 18,348 | 111 (79/28/4) |
| *Artemisia frigida* | JX293720 | 151,076 | 37.48 | 82,740 | 24,972 | 18,392 | 113 (80/29/4) |
| *Artemisia gmelinii* | KU736962 | 151,318 | 37.42 | 83,061 | 24,961 | 18,335 | 114 (80/30/4) |
| *Artemisia freyniana f. discolor* | MG951487 | 151,275 | 37.45 | 82,965 | 24,985 | 18,340 | 114 (80/30/4) |
| *Artemisia feddei* | MG951486 | 151,112 | 37.49 | 82,878 | 24,958 | 18,318 | 114 (80/30/4) |
| *Artemisia stolonifera* | MG951500 | 151,144 | 37.47 | 82,878 | 24,960 | 18,346 | 114 (80/30/4) |
| *Artemisia montana* | KF887960 | 151,130 | 37.48 | 82,873 | 24,959 | 18,339 | 113 (80/29/4) |
| *Artemisia rubripes* | MG951496 | 151,133 | 37.48 | 82,874 | 24,959 | 18,341 | 114 (80/30/4) |
| *Artemisia argyi* | KM386991 | 151,192 | 37.46 | 82,930 | 24,959 | 18,344 | 112 (79/29/4) |
| *Artemisia princeps* | MG951495 | 151,193 | 37.46 | 82,932 | 24,959 | 18,343 | 114 (80/30/4) |

Gene counts are shown as unique genes; values in parentheses indicate protein-coding genes, tRNA genes, and rRNA genes, respectively.

**Table S4. Complete gene annotation of the chloroplast genome of *Artemisia schmidtiana*.**

| **Gene name** | **Gene type** | **Functional category** | **Region** | **Genomic coordinates (bp)** | **Intron status** | **Duplicated in IR** |
| --- | --- | --- | --- | --- | --- | --- |
| *trnH-GUG* | tRNA | Transfer RNA | LSC | complement(9..83) | No | No |
| *psbA* | PCG | Photosystem II | LSC | complement(465..1526) | No | No |
| *trnK-UUU* | tRNA | Transfer RNA | LSC | complement(1726..4346) | Yes (cis-spliced) | No |
| *matK* | PCG | Maturase | LSC | complement(2063..3580) | No | No |
| *rps16* | PCG | Small ribosomal protein | LSC | complement(5204..6287) | Yes (cis-spliced) | No |
| *trnQ-UUG* | tRNA | Transfer RNA | LSC | complement(7188..7259) | No | No |
| *psbK* | PCG | Photosystem II | LSC | 7615..7794 | No | No |
| *psbI* | PCG | Photosystem II | LSC | 8188..8298 | No | No |
| *trnS-GCU* | tRNA | Transfer RNA | LSC | complement(8430..8517) | No | No |
| *trnC-GCA* | tRNA | Transfer RNA | LSC | 9260..9340 | No | No |
| *petN* | PCG | Cytochrome b/f complex | LSC | 9911..10000 | No | No |
| *psbM* | PCG | Photosystem II | LSC | complement(10528..10632) | No | No |
| *trnD-GUC* | tRNA | Transfer RNA | LSC | complement(11307..11388) | No | No |
| *trnY-GUA* | tRNA | Transfer RNA | LSC | complement(11501..11584) | No | No |
| *trnE-UUC* | tRNA | Transfer RNA | LSC | complement(11786..11857) | No | No |
| *rpoB* | PCG | RNA polymerase | LSC | 12730..15912 | No | No |
| *rpoC1* | PCG | RNA polymerase | LSC | 15939..18740 | Yes (cis-spliced) | No |
| *rpoC2* | PCG | RNA polymerase | LSC | 18848..23005 | No | No |
| *rps2* | PCG | Small ribosomal protein | LSC | 23259..23969 | No | No |
| *atpI* | PCG | ATP synthase | LSC | 24185..24928 | No | No |
| *atpH* | PCG | ATP synthase | LSC | 26065..26310 | No | No |
| *atpF* | PCG | ATP synthase | LSC | 26679..27932 | Yes (cis-spliced) | No |
| *atpA* | PCG | ATP synthase | LSC | 28002..29528 | No | No |
| *trnR-UCU* | tRNA | Transfer RNA | LSC | complement(29668..29739) | No | No |
| *trnG-UCC* | tRNA | Transfer RNA | LSC; LSC | complement(29965..30763); 35613..35683 | Yes (cis-spliced) | No |
| *psbD* | PCG | Photosystem II | LSC | 32041..33102 | No | No |
| *psbC* | PCG | Photosystem II | LSC | 33050..34471 | No | No |
| *trnS-UGA* | tRNA | Transfer RNA | LSC | complement(34680..34762) | No | No |
| *psbZ* | PCG | Photosystem II | LSC | 35109..35297 | No | No |
| *trnfM-CAU* | tRNA | Transfer RNA | LSC | complement(35867..35940) | No | No |
| *rps14* | PCG | Small ribosomal protein | LSC | complement(36095..36397) | No | No |
| *psaB* | PCG | Photosystem I | LSC | complement(36531..38735) | No | No |
| *psaA* | PCG | Photosystem I | LSC | complement(38761..41013) | No | No |
| *ycf3* | PCG | Conserved open reading frame | LSC | complement(41733..43681) | Yes (cis-spliced) | No |
| *trnS-GGA* | tRNA | Transfer RNA | LSC | 44523..44608 | No | No |
| *rps4* | PCG | Small ribosomal protein | LSC | complement(44937..45542) | No | No |
| *trnT-UGU* | tRNA | Transfer RNA | LSC | complement(45901..45973) | No | No |
| *trnL-UAA* | tRNA | Transfer RNA | LSC | 46482..46994 | Yes (cis-spliced) | No |
| *trnF-GAA* | tRNA | Transfer RNA | LSC | 47358..47427 | No | No |
| *ndhJ* | PCG | NADH dehydrogenase | LSC | complement(48126..48602) | No | No |
| *ndhK* | PCG | NADH dehydrogenase | LSC | complement(48705..49382) | No | No |
| *ndhC* | PCG | NADH dehydrogenase | LSC | complement(49435..49797) | No | No |
| *trnV-UAC* | tRNA | Transfer RNA | LSC | complement(50943..51590) | Yes (cis-spliced) | No |
| *trnM-CAU* | tRNA | Transfer RNA | LSC | 51766..51838 | No | No |
| *atpE* | PCG | ATP synthase | LSC | complement(52037..52438) | No | No |
| *atpB* | PCG | ATP synthase | LSC | complement(52435..53913) | No | No |
| *rbcL* | PCG | Rubisco | LSC | 54668..56125 | No | No |
| *accD* | PCG | Acetyl-CoA carboxylase | LSC | 56630..58132 | No | No |
| *psaI* | PCG | Photosystem I | LSC | 58619..58729 | No | No |
| *ycf4* | PCG | Conserved open reading frame | LSC | 59102..59656 | No | No |
| *cemA* | PCG | Envelope membrane protein | LSC | 59931..60620 | No | No |
| *petA* | PCG | Cytochrome b/f complex | LSC | 60856..61818 | No | No |
| *psbJ* | PCG | Photosystem II | LSC | complement(62594..62716) | No | No |
| *psbL* | PCG | Photosystem II | LSC | complement(62865..62981) | No | No |
| *psbF* | PCG | Photosystem II | LSC | complement(63004..63123) | No | No |
| *psbE* | PCG | Photosystem II | LSC | complement(63133..63384) | No | No |
| *petL* | PCG | Cytochrome b/f complex | LSC | 64588..64683 | No | No |
| *petG* | PCG | Cytochrome b/f complex | LSC | 64854..64967 | No | No |
| *trnW-CCA* | tRNA | Transfer RNA | LSC | complement(65085..65158) | No | No |
| *trnP-UGG* | tRNA | Transfer RNA | LSC | complement(65342..65415) | No | No |
| *psaJ* | PCG | Photosystem I | LSC | 65724..65858 | No | No |
| *rpl33* | PCG | Large ribosomal protein | LSC | 66298..66498 | No | No |
| *rps18* | PCG | Small ribosomal protein | LSC | 66670..66975 | No | No |
| *rpl20* | PCG | Large ribosomal protein | LSC | complement(67239..67619) | No | No |
| *rps12* | PCG | Small ribosomal protein | LSC/IRb; LSC/IRa | join(complement(68346..68459), complement(96532..96763), complement(95971..95996)); join(complement(68346..68459), 137076..137307, 137843..137868) | Yes (trans-spliced) | Yes |
| *clpP* | PCG | Protease | LSC | complement(68614..70637) | Yes (cis-spliced) | No |
| *psbB* | PCG | Photosystem II | LSC | 71086..72612 | No | No |
| *psbT* | PCG | Photosystem II | LSC | 72804..72905 | No | No |
| *psbH* | PCG | Photosystem II | LSC | 73215..73436 | No | No |
| *petB* | PCG | Cytochrome b/f complex | LSC | 73561..74954 | Yes (cis-spliced) | No |
| *petD* | PCG | Cytochrome b/f complex | LSC | 75143..76305 | Yes (cis-spliced) | No |
| *rpoA* | PCG | RNA polymerase | LSC | complement(76515..77522) | No | No |
| *rps11* | PCG | Small ribosomal protein | LSC | complement(77602..78012) | No | No |
| *rpl36* | PCG | Large ribosomal protein | LSC | complement(78135..78248) | No | No |
| *infA* | PCG | Translation initiation factor | LSC | complement(78355..78588) | No | No |
| *rps8* | PCG | Small ribosomal protein | LSC | complement(78710..79114) | No | No |
| *rpl14* | PCG | Large ribosomal protein | LSC | complement(79299..79667) | No | No |
| *rpl16* | PCG | Large ribosomal protein | LSC | complement(79786..81211) | Yes (cis-spliced) | No |
| *rps3* | PCG | Small ribosomal protein | LSC | complement(81371..82027) | No | No |
| *rpl22* | PCG | Large ribosomal protein | LSC | complement(82012..82479) | No | No |
| *rps19* | PCG | Small ribosomal protein | LSC/IRb | complement(82588..82866) | No | No |
| *rpl2* | PCG | Large ribosomal protein | IRb; IRa | complement(82923..84412); 149427..150916 | Yes (cis-spliced) | Yes |
| *rpl23* | PCG | Large ribosomal protein | IRb; IRa | complement(84431..84712); 149127..149408 | No | Yes |
| *trnI-CAU* | tRNA | Transfer RNA | IRb; IRa | complement(84878..84951); 148888..148961 | No | Yes |
| *ycf2* | PCG | Conserved open reading frame | IRb; IRa | 85063..91902; complement(141937..148776) | No | Yes |
| *trnL-CAA* | tRNA | Transfer RNA | IRb; IRa | complement(92309..92389); 141450..141530 | No | Yes |
| *ndhB* | PCG | NADH dehydrogenase | IRb; IRa | complement(92952..95154); 138685..140887 | Yes (cis-spliced) | Yes |
| *rps7* | PCG | Small ribosomal protein | IRb; IRa | complement(95450..95917); 137922..138389 | No | Yes |
| *ycf15* | PCG | Conserved open reading frame | IRb | complement(97720..97911) | No | No |
| *trnV-GAC* | tRNA | Transfer RNA | IRb; IRa | 98608..98679; complement(135160..135231) | No | Yes |
| *rrn16* | rRNA | Ribosomal RNA | IRb; IRa | 98906..100396; complement(133443..134932) | No | Yes |
| *trnI-GAU* | tRNA | Transfer RNA | IRb; IRa | 100690..101542; complement(132297..133149) | Yes (cis-spliced) | Yes |
| *trnA-UGC* | tRNA | Transfer RNA | IRb; IRa | 101607..102491; complement(131348..132232) | Yes (cis-spliced) | Yes |
| *rrn23* | rRNA | Ribosomal RNA | IRb; IRa | 102644..105453; complement(128386..131195) | No | Yes |
| *rrn4.5* | rRNA | Ribosomal RNA | IRb; IRa | 105552..105654; complement(128185..128287) | No | Yes |
| *rrn5* | rRNA | Ribosomal RNA | IRb; IRa | 105900..106020; complement(127819..127939) | No | Yes |
| *trnR-ACG* | tRNA | Transfer RNA | IRb; IRa | 106242..106315; complement(127524..127597) | No | Yes |
| *trnN-GUU* | tRNA | Transfer RNA | IRb; IRa | complement(106788..106860); 126979..127051 | No | Yes |
| *ndhF* | PCG | NADH dehydrogenase | SSC | complement(107807..110032) | No | No |
| *rpl32* | PCG | Large ribosomal protein | SSC | 111028..111192 | No | No |
| *trnL-UAG* | tRNA | Transfer RNA | SSC | 112068..112147 | No | No |
| *ccsA* | PCG | Cytochrome c synthesis | SSC | 112255..113229 | No | No |
| *ndhD* | PCG | NADH dehydrogenase | SSC | complement(113431..114999) | No | No |
| *psaC* | PCG | Photosystem I | SSC | complement(115051..115296) | No | No |
| *ndhE* | PCG | NADH dehydrogenase | SSC | complement(115530..115835) | No | No |
| *ndhG* | PCG | NADH dehydrogenase | SSC | complement(116037..116567) | No | No |
| *ndhI* | PCG | NADH dehydrogenase | SSC | complement(116926..117426) | No | No |
| *ndhA* | PCG | NADH dehydrogenase | SSC | complement(117503..119656) | Yes (cis-spliced) | No |
| *ndhH* | PCG | NADH dehydrogenase | SSC | complement(119658..120839) | No | No |
| *rps15* | PCG | Small ribosomal protein | SSC | complement(120931..121209) | No | No |
| *ycf1* | PCG | Conserved open reading frame | SSC/IRa | complement(121624..126651) | No | No |

Coordinates are 1-based and refer to the GenBank record PX309299. The table lists 111 unique genes (79 PCGs, 28 tRNA genes, and 4 rRNA genes). For genes duplicated in the IR regions, both IRb and IRa coordinates are provided in the same row; compound coordinates indicate exon-containing genes.
